# Dengue and Chikungunya Fever among Viral Diseases in Outpatient Febrile Children in Kilosa District Hospital, Tanzania

**DOI:** 10.1371/journal.pntd.0003335

**Published:** 2014-11-20

**Authors:** Beatrice Chipwaza, Joseph P. Mugasa, Majige Selemani, Mbaraka Amuri, Fausta Mosha, Steve D. Ngatunga, Paul S. Gwakisa

**Affiliations:** 1 Nelson Mandela African Institution of Science and Technology, School of Life Sciences and Bioengineering, Arusha, Tanzania; 2 Ifakara Health Institute, Ifakara, Tanzania; 3 National Institute for Medical Research, Amani Medical Research Centre, Muheza, Tanga, Tanzania; 4 Ifakara Health Institute, Dar es Salaam, Tanzania; 5 Jhpiego, Dar-es-Salaam, Tanzania; 6 National Health Laboratory, Ministry of Health and Social Welfare, Dar-es-salaam, Tanzania; 7 National Institute for Medical Research, Kilosa, Morogoro, Tanzania; 8 Genome Science Centre and Department of Veterinary Microbiology and Parasitology, Sokoine University of Agriculture, Morogoro, Tanzania; Stanford University School of Medicine, United States of America

## Abstract

**Introduction:**

Viral etiologies of fever, including dengue, Chikungunya, influenza, rota and adeno viruses, cause major disease burden in tropical and subtropical countries. The lack of diagnostic facilities in developing countries leads to failure to estimate the true burden of such illnesses, and generally the diseases are underreported. These diseases may have similar symptoms with other causes of acute febrile illnesses including malaria and hence clinical diagnosis without laboratory tests can be difficult. This study aimed to identify viral etiologies as a cause of fever in children and their co-infections with malaria.

**Methods:**

A cross sectional study was conducted for 6 months at Kilosa district hospital, Tanzania. The participants were febrile children aged 2–13 years presented at the outpatient department. Diagnostic tests such as IgM and IgG ELISA, and PCR were used.

**Results:**

A total of 364 patients were enrolled, of these 83(22.8%) had malaria parasites, 76 (20.9%) had presumptive acute dengue infection and among those, 29(38.2%) were confirmed cases. Dengue was more likely to occur in children ≥ 5 years than in <5 years (OR 2.28, 95% CI: 1.35–3.86). Presumptive acute Chikungunya infection was identified in 17(4.7%) of patients. We observed no presenting symptoms that distinguished patients with Chikungunya infection from those with dengue infection or malaria. Co-infections between malaria and Chikungunya, malaria and dengue fever as well as Chikungunya and dengue were detected. Most patients with Chikungunya and dengue infections were treated with antibacterials. Furthermore, our results revealed that 5(5.2%) of patients had influenza virus while 5(12.8%) had rotavirus and 2(5.1%) had adenovirus.

**Conclusion:**

Our results suggest that even though viral diseases are a major public health concern, they are not given due recognition as a cause of fever in febrile patients. Emphasis on laboratory diagnostic tests for proper diagnosis and management of febrile patients is recommended.

## Author Summary

Viral etiologies of fever, such as dengue, Chikungunya, influenza, rotaviruses, and adenoviruses contribute to substantial morbidity and mortality particularly in children in the tropical and subtropical countries. Despite their importance, the true burden of viral etiologies of fever in Tanzania is not known, and in most cases these diseases are unrecognized and treated as malaria. This study aimed to identify viral etiologies as a cause of fever in children and co-infections among viral diseases and malaria. A total of 364 febrile children were enrolled, of these, 83 (22.8%) had malaria, 76(20.9%) had presumptive acute dengue, among those, 29(38.2%) were confirmed cases. Dengue was more likely to occur in children aged ≥ 5 years than in <5 years (OR 2.28, 95% CI: 1.35–3.86). Presumptive acute Chikungunya infection was found in 17(4.7%) of patients. Co-infections between malaria and Chikungunya, malaria and dengue as well as Chikungunya and dengue were observed. Furthermore, 5(5.2%) of the patients had influenza virus, 5(12.8%) had rotavirus and 2(5.1%) had adenovirus. Knowledge on occurrence of these viral etiologies of febrile illnesses will alert clinicians to be more responsive towards viral infectious agents as potential causes of febrile illnesses in the routine diagnosis and management of febrile patients.

## Introduction

Febrile illness is one of the most common reasons for seeking medical attention. Febrile illnesses which are caused by different etiological agents are the leading cause of morbidity and mortality particularly in developing countries [1]. For many years, malaria has been the foremost cause of fever in children and it contributed for substantial under five mortalities in sub-Saharan Africa [2]. In recent years, there has been achievement in malaria control strategies which has led to a reduction of malaria prevalence as well as malaria transmission, morbidity and mortality [3]–[5]. However, despite the decrease of malaria, fever is still a major complaint among patients and thus highlights the importance of non malarial causes of fever. Recent studies have demonstrated that a high proportion of fevers are due to non-malaria febrile illnesses [6], [7]. Several non-malarial febrile illnesses due to different etiological agents have been reported in Tanzania. Diseases such as urinary tract infections, respiratory tract infections, and typhoid fever are among the most common particularly in children [7].

Apart from bacterial diseases viral etiologies, namely dengue fever virus (DENV), Chikungunya virus (CHIKV), influenza virus, rota- and adeno- viruses have also been reported [8], [9]. DENV and CHIKV are arthropod-borne viruses that are transmitted to humans by Aedes mosquitoes and cause major disease burden in tropical and subtropical countries worldwide. Dengue fever is caused by a virus of the genus Flavivirus and there are four serotypes (DENV 1-4) while Chikungunya is caused by an alpha virus [10], [11]. In addition, influenza virus, rota- and adeno-viruses cause significant morbidity and mortality in children [12], [13]. For instance, rotavirus infection contributes to more than a third of diarrhea among children under five years of age and it is approximated to cause more than 8,100 deaths of Tanzanian children under five in each year [14]. These diseases are a major public health concern and have impacts on economies [15], [16].

Lack of diagnostic facilities in most health care facilities makes it difficult to estimate the true prevalence of such illnesses and in most cases the diseases are underreported [17]. In addition, in the absence of diagnostic facilities including malaria Rapid Diagnostic Tests (mRDTs), the ultimate diagnosis of a febrile illness is reached mostly based on clinical ground and thus most fever cases are presumed to be malaria and treated with unwarranted anti-malarial drugs [18]. In the absence of a supportive laboratory test malaria over diagnosis is a likely manifestation [19]. Furthermore, patients with non-malaria febrile illnesses such as dengue and Chikungunya may be presented with similar manifestations thus laboratory confirmation is essential.

Even though there are reported cases of DENV and CHIKV infections in Northern Tanzania and a recent outbreak of dengue fever in some regions [8] very few studies have been conducted to determine the prevalence and the distribution of these diseases [6]. Also, information on the prevalence of viral diseases such as influenza virus, rotavirus and adenovirus is limited. Furthermore, identification of etiologies of fever cases which occur concurrently with malaria is not commonly practiced in most health facilities in Tanzania. Therefore, treatment with anti-malarial drugs alone results into incomplete therapy and possibly may cause deaths due to undiagnosed infections. This study intended to contribute in bridging the information gap through determining viral etiologies as a cause of fever in children. The study also evaluated viral diseases which occur concurrently with malaria and co-infections between viral febrile illnesses. Knowledge on occurrence of viral etiologies of febrile illnesses, occurring either as isolated cases or as concomitant with malaria will make clinicians more responsive to consider viral infectious agents as important causes of febrile illnesses in routine diagnosis and management of febrile patients. Therefore, this will assist in the management of febrile patients and ultimately reduce the overuse of anti-malarials. In addition, finding of viral etiologies as a cause of non-malaria fevers will provide evidence to policy makers and national disease control programs on better ways for management of such illnesses.

## Materials and Methods

### Study area

**Figure 1:**
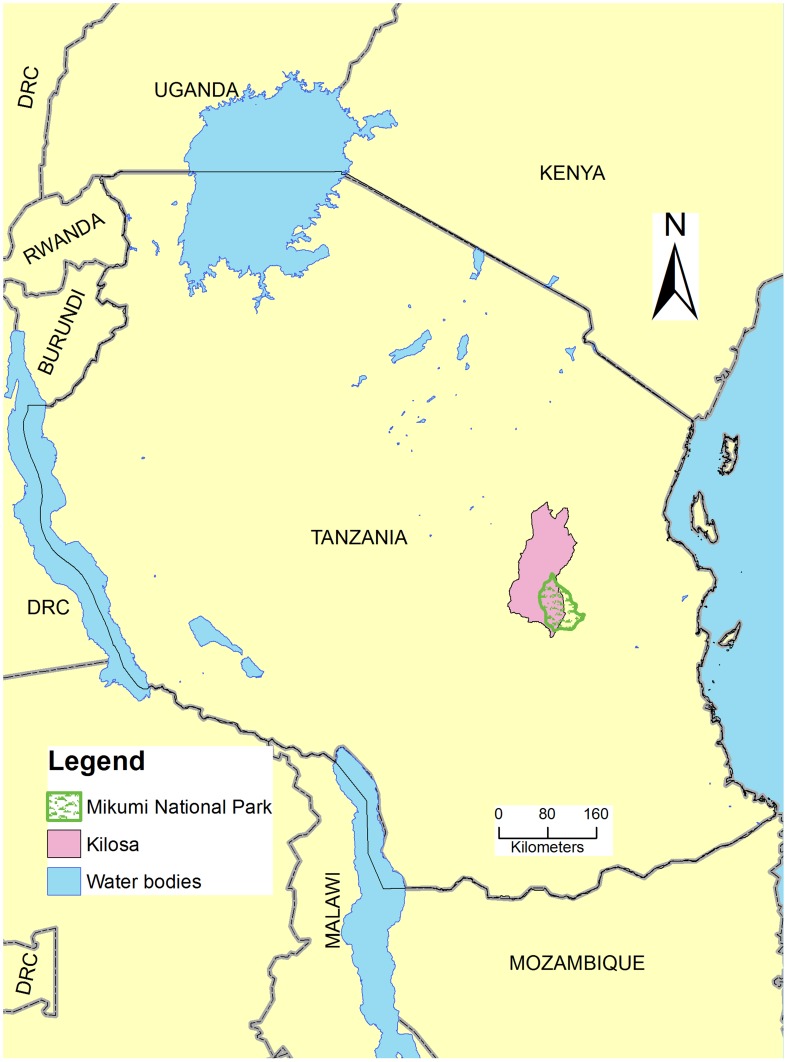
Map of Tanzania showing the study area.

The study was conducted at Kilosa district hospital in Morogoro region, Tanzania. Kilosa district borders with Tanga and Manyara regions to the north and Mvomero district and Mikumi National Park to the east. On the western border are Dodoma and Iringa regions whereas to the south it borders with Kilombero district (Figure 1). The district lies between latitudes 6° south and 8° south and longitudes 36° 30′ east and 38° east. The area has semi humid climate with an average rainfall of 800 mm annually. The short rains start in November and end in January followed by heavy rainfall between March and May. The district experiences a dry season from June to October and the average annual temperature is 24.6°C. The district has an area of 14,245 square kilometers and a population of 438,175 people [20]. The proportion of children under the age of 5 and 10 years is 65,654 and 62,235, respectively [21]. According to 2005/2006 statistics, the infant mortality rate and under-five mortality rate in Kilosa district were 112 per 1000 live births and 166 per 1000 live births respectively [22] while in 2012/2013 the rates were 74 and 192 per 1000 live births respectively [23]. The main economic activities in the district are crop production and livestock keeping.

Administratively, the district is divided into 9 divisions, 37 wards and 164 villages [24]. In terms of health care services, it has 71 health facilities and among these, there are 3 hospitals, 7 health centers and 61 dispensaries [25]. Kilosa district hospital acts as referral for the primary health care facilities in the district [26]. Kilosa district is an area with holoendemic malaria transmission [27]. In 2007, malaria accounted for about 55.5% of the total outpatient cases, and 60% of the total deaths among under-fives admitted to the health facilities [28]. However, in 2011–2012 malaria prevalence was estimated to be 13% [29]. The common non-malaria febrile illnesses that have been reported in Kilosa district include acute respiratory diseases, UTIs and typhoid fever [30]. In addition, outbreaks of Rift Valley fever (RVF) have been reported in the district and the most recent outbreak, which occurred in 2006/07 was associated with morbidity in humans and livestock as well as significant mortalities and abortions in livestock [31], [32]. However, there are no reports describing the occurrence of arbovirus infections, such as DENV disease in the district. With regards to yellow fever, no cases in humans or non-human primates have been reported in Tanzania and children are not vaccinated against this disease [33] but rather yellow fever vaccination is mandatory to international travelers.

### Study design and participants

This was a cross sectional study which was conducted for 6 months. The study was carried out during rainy season (March–May 2013) and dry season (August–October 2013). The study participants were recruited from children who presented at the outpatient department (OPD) at Kilosa district hospital. The inclusion criteria were children aged between 2–13 years on the day of enrollment with measured axillary or rectal temperature >37.5°C or ≥38°C, respectively. The exclusion criteria were children with chronic diseases or severe illnesses, which required immediate or inpatient treatment. On average a maximum of two children were recruited per day.

### Clinical examination and sample collection

The clinical history, including history of fever, and physical examination were performed by a trained clinical officer of the respective hospital. The physician recorded symptoms associated with febrile illness and findings obtained after performing standard clinical examination of the patients. The clinical diagnosis and patient management were done according to the local standard of care and were recorded on a standard assessment form. Five milliliters of venous blood were collected aseptically into tubes with ethylene-diamine-tetraacetic-acid (EDTA) and plain tubes from all enrolled patients. Nasal and throat swabs were taken in viral transport medium (VTM) from children with nasal discharge. In addition, stool sample was collected from patients with diarrhea. All samples were temporarily stored at −20°C at Kilosa district hospital before being transported to Ifakara Health Institute (IHI) where they were kept at −80°C until analyzed.

### Laboratory analysis

Laboratory evaluation was based on the reflection of viral etiologies of febrile illnesses that might occur in the study area. Therefore, patients were screened against CHIKV, DENV, influenza virus, rota- and adeno- viruses' infections. In addition, all patients were checked for malaria parasites. Bacterial causes of fever were also investigated at a later point, and detailed results thereof will be presented in a separate paper.

#### Malaria

The examination of malaria parasites was initially performed by the laboratory technician from the district hospital. Thick and thin blood films were stained with Giemsa and examined for blood parasites by microscopy. Each slide was read independently by a second experienced microscopists from IHI and any discrepancies were resolved by a third microscopist. The parasite density was determined by standard methods [34].

#### Serologic testing

The serologic tests were conducted at IHI laboratory at Ifakara in Morogoro region, Tanzania. Serum sample from each patient was tested for anti-DENV and anti-CHIKV immunoglobulin M (IgM) and immunoglobulin G (IgG) antibodies using enzyme-linked immunosorbent assay (ELISA) (both NovaTec Immundiagnostica GmbH, Germany) according to manufacturer instructions. The sensitivity and specificity of these tests, as determined by the manufacturers, are shown in Table S1.

#### Detection of Rotavirus and Adenovirus

The presence of Rotaviruses and/or Adenoviruses was assessed by testing stool samples from children with diarrhea. The detection was performed by RIDA QUICK Rotavirus/Adenovirus Combi (R-Biopharm AG, Darmstadt, Germany). This is a single-step immunochromatographic lateral-flow test, which detects antigens from stool samples (sensitivity and specificity in Table S1). The test was performed according to the manufacturer's instructions. Briefly, each stool sample was mixed with extraction buffer and homogenized on a vortex mixer. The stool sample was allowed to settle and 200µl of clear supernatant was added into the round opening of the cassette and the results were read after 5 minutes.

#### Real time PCR for influenza and DENV

Real time PCR (RT-PCR) was conducted at the National Health Laboratory Quality Assurance Training Center (NHLQATC), Dar-es-Salaam, Tanzania. RNA was extracted from plasma samples using the QIAamp Viral RNA Mini kit (QIAGEN, Hilden, Germany). Center for Disease Control and Prevention (CDC) DENV-1-4- Real-Time RT-PCR assay and CDC Influenza virus RT-PCR influenza A/B kits were used for amplification of DENV and influenza virus respectively according to manufacturer instructions [35], [36]. Plasma samples from patients with positive anti-dengue IgM antibodies were further tested by DENV RT-PCR. The RT-PCR for both DENV and influenza viruses was done in a total reaction volume of 25 µL. Reverse transcription was performed using Invitrogen Superscript III First Strand Synthesis System (Carlsbad, CA, USA) and RT-PCR was carried out by Applied Biosystems 7500 fast RT-PCR system (San Mateo, CA, USA). DENV RT-PCR assay was run in a singleplex i.e. each DENV serotype was detected in a separate reaction. The conditions for DENV RT-PCR were 50°C for 30 minutes, 95°C for 2 minutes and 45 cycles at 95°C for 15 seconds and 60°C for 1 minute. For influenza virus, PCR conditions were 50°C for 30 minutes, 95°C for 2 minutes and 45 cycles at 95°C for 15 seconds and 60°C for 30 seconds. Due to lack of positive controls, RT-PCR for CHIKV was not was not performed.

### Case definitions

Fever was classified as mild if temperature was 37.5–38.3°C, moderate at 38.4–39.4°C and high at >39.5°C. Confirmed DENV/influenza virus was defined as a positive RT-PCR result for DENV and influenza virus. Presumptive acute DENV/CHIKV was defined as detection of anti-DENV/CHIKV IgM by ELISA. Probable prior exposure was defined as positive anti-DENV/CHIKV IgG by ELISA [37].

### Data management and statistical analysis

Data were entered into an Access database by an experienced data clerk. The database was designed to ascertain validation rules of each data field. The verification of data entry and data cleaning was done to ensure that clinical data and laboratory findings are matched. The cleaned data were then transferred to STATA using Stat transfer version 9 and statistical analyses were performed using STATA software (version 11; Stata Corp., TX USA). Pearson's Chi-Square test was used to determine the association between categorical variables. An alpha level of 0.05 was used for all tests of statistical significance.

### Ethics statement

This study was approved by Institutional Review Board of Ifakara Health Institute (IHI/IRB/No: 01-2013) and Medical Research Coordinating Committee of Tanzania's National Institute for Medical Research (NIMR/HQ/R.8a/Vol.1X/1472). Children under 12 years had written informed consent given from a parent or guardian. In addition a verbal assent was also obtained from children aged 7–12 years. However, children above 12 years provided their own written informed consent which was accompanied by the consent of a parent or guardian.

## Results

The total number of febrile children enrolled in the study was 364, including 186 (51.1%) males, 178 (48.9%) females. The patients aged less than 5 years were 205(56.3%) and above 5 years were 159 (43.7%). One hundred eighty four patients were enrolled during the rainy season while 180 during the dry season. The majority of patients (75.6%) had mild fever during the time of enrollment and only 5% had high fever. The duration of fever (history of fever) in many patients was between 1–7 days while few had fever for more than 7 days. Before recruitment 103 (28.3%) of the patients had received anti-malarials and antibacterials whereas 261 (71.7%) neither used anti-malarials nor antibacterials. The patients' demographic characteristics, the enrollment season and temperature are summarized in Table 1.

**Table 1:** Demographic characteristics, enrollment season and body temperature for all participants.

Category	Subcategory	n	%
**Gender**	Male	186	51.1
	Female	178	48.9
**Age**	<5 years	205	56.3
	≥ 5 years	159	43.7
**Season**	Rainy	184	50.6
	Dry	180	49.4
**Temperature**	Mild	275	75.6
	Moderate	84	23.1
	High	5	1.4
**Duration of fever**	1–3 days	181	49.7
	4–7 days	175	48.1
	>7 days	5	1.4
	Unknown	3	0.8
**Recent therapy**	No therapy	261	71.7
	Recent therapy	103	28.3
	Anti-malarials	76	73.8
	Antibacterials	11	10.7
	Anti-malarial + antibacterials	16	15.5

### Malaria

Among the enrolled patients, 83 (22.8%) had malaria including 44 (23.7%) males and 39 (21.9%) females. Malaria was detected in 47 (22.9%) children under five years while in children above five years it was detected in 36 (22.6%), Table 2. Despite the fact that there was no statistical significance between the prevalence of malaria and age, sex and season, the prevalence of malaria was higher during the dry season than during rainy season (Odds ratio [OR] 1.56, 95% CI: 0.95–2.58), Table 3. The main symptoms/clinical signs presented by patients with malaria were vomiting 29 (34.9%), abdominal pain 14 (16.9%), cough 7 (8.4%), diarrhea 5 (6.0%), headache 4 (4.8%) and joint pain 3 (3.7%), Table 2.

**Table 2:** Proportions of patients with Chikungunya, dengue, the associated symptoms, provisional diagnosis and treatment given.

		Malaria parasites		Presumptive acute CHIKV		Prior CHIKV exposure		Presumptive acute DENV		Prior DENV exposure		Confirmed acute DENV	
Category	Subcategory	n/N	%	n/N	%	n/N	%	n/N	%	n/N	%	n/N	%
**Overall prevalence**		83/364	22.8	17/364	4.7	5/364	1.4	76/364	20.9	24/364	6.6	29/76	38.2
**Gender**	Male	44/186	23.7	8/186	4.3	3/186	1.6	34/186	18.3	17/186	9.1	15/34	44.1
	Female	39/178	21.9	9/178	5.1	2/178	1.1	42/178	23.6	7/178	3.9	14/42	33.3
**Age**	<5 yrs	47/205	22.9	11/205	5.4	2/205	1.0	30/205	14.6	8/205	3.9	5/30	16.7
	≥5 yrs	36/159	22.6	6/159	3.8	3/159	1.9	46/159	28.9	16/159	10.1	24/46	52.2
**Season**	Rainy season	35/184	19.0	6/184	3.3	1/184	0.5	43/184	23.4	16/184	8.7	9/43	20.9
	Dry season	48/180	26.7	11/180	6.1	4/180	2.2	33/180	18.3	8/180	4.4	20/33	60.6
**Duration of fever**	1–3 days	43/83	51.8	10/17	58.8	3/5	60	30/76	39.5	5/24	20.8	11/29	37.9
	4–7 days	38/83	45.8	7/17	41.2	2/5	40	43/76	56.6	18/24	75.0	17/29	58.6
	> 7 days	2/83	2.4	0	0	0	0	1/76	1.3	1/24	4.2	1/29	3.4
	Unknown	0	0	0	0	0	0	2/76	2.6	0	0	0	0
**Symptoms**	Headache	4/83	4.8	1/17	5.9	1/5	20.0	7/76	9.2	1/24	4.2	4/29	13.8
	Abdominal pain	14/83	16.9	2/17	11.8	1/5	20.0	6/76	7.9	2/24	8.3	4/29	13.8
	Rashes	4/83	4.8	3/17	17.7	1/5	20.0	7/76	9.2	2/24	8.3	3/29	10.3
	Diarrhea	5/83	6.0	1/17	5.9	1/5	20.0	5/76	6.6	1/24	4.2	2/29	6.9
	Vomiting	29/83	34.9	0	0	1/5	20.0	9/76	11.8	7/24	29.2	7/29	24.1
	Joint pain	3/83	3.7	1/17	5.9	0	20.0	3/76	4.0	0	0	2/29	6.9
	Cough	7/83	8.4	7/17	41.2	2/5	40.0	14/76)	18.4	1/24	4.2	4/29	13.8
	URTα	1/83	1.2	0	0	0	0	5/76	6.6	3/24	12.5	2/29	6.9
	LRT ^¶^	2/83	2.4	7/17	41.2	1/5	20.0	15/76	19.7	2/24	8.3	5/29	17.2
	Other^*^	5/83	6.0	1/17	5.9	0	0	5/76	6.6	3/24	12.5	1/29	3.4
**Provisional diagnosis**	Malaria	81/83	97.6	2/17	11.8	1/5	20.0	18/76	23.7	6/24	25.0	8/29	27.6
	Urinary tract infections	25/83	30.1	10/17	58.8	2/5	40.0	42/76	55.3	6/24	25.0	15/29	51.7
	Pneumonia	3/83	3.6	7/17	41.2	1/5	20.0	16/76	21.1	0	0	4/29	13.8
	Sore throat	0	0	0	0	0	0	2/76	2.6	2/24	8.3	1/29	3.4
	Other^§^	1/83	1.2	1/17	5.9	2/5	40.0	11/76	14.5	7/24	29.2	5/29	17.2
**Treatment**	Anti-malarials	79/83	95.2	2/17	11.8	1/5	20.0	18/76	23.7	6/24	25.0	8/29	27.6
	Antibacterials	34/83	41.0	15/17	88.2	4/5	80.0	68/76	89.5	17/24	70.8	24/29	82.8
	Antipyretics	72/83	86.8	8/17	47.1	3/5	60.0	51/76	67.1	18/24	75.0	22/29	75.9
	Other drugs^#^	1/83	1.2	2/17	11.8	1/5	20.0	11/76	14.5	8/24	33.3	6/29	20.7

α Upper respiratory tract symptoms; ^¶^ Lower respiratory tract symptoms; *Backache, nose bleeding, loss of appetite, neck pain, swelling feet, difficulty breathing and eye pain; ^§^ Chiken pox, boils, otitis media, enteric fever, herpes simplex, rhinitis and dysentery; ^#^ Paediatric zinc, prednisolone, vitamins and ORS (Oral salts).

**Table 3:** Associations between Malaria, CHIKV and DENV infections with sex, age and season.

	Category	Subcategory	Odds Ratio	95% CI	p-value
**Malaria**	Sex	Male	1.00		
		Female	0.90	0.55–1.48	0.683
	Age	<5 years	1.00		
		≥ 5 years	1.06	0.64–1.76	0.811
	Season	Wet season	1.00		
		Dry season	1.56	0.95–2.58	0.081
**Presumptive acute CHIKV**	Sex	Male	1.00		
		Female	1.20	0.45–3.19	0.717
	Age	<5 years	1.00		
		≥ 5 years	0.76	0.27–2.12	0.597
	Season	Wet season	1.00		
		Dry season	1.85	0.66–5.17	0.242
**Presumptive acute DENV**	Sex	Male	1.00		
		Female	1.34	0.80–2.24	0.268
	Age	<5 years	1.00		
		≥ 5 years	2.28	1.35–3.86	0.002
	Season	Wet season	1.00		
		Dry season	0.84	0.50–1.41	0.507
**Probable prior DENV exposure**	Sex	Male	1.00		
		Female	0.38	0.15–0.95	0.038
	Age	<5 years	1.00		
		≥ 5 years	2.69	1.10–6.56	0.030
	Season	Wet season	1.00		
		Dry season	0.56	0.23–1.36	0.197
**Confirmed acute DENV**	Sex	Male	1.00		
		Female	0.35	0.11–1.15	0.083
	Age	<5 years	1.00		
		≥ 5 years	6.51	1.78–23.87	0.005
	Season	Wet season	1.00		
		Dry season	5.03	1.68–15.01	0.004

### Dengue virus

Seventy six (20.9%) of febrile children met the definition for presumptive acute DENV infection, of these, 34 (18.3%) were males and 42 (23.6%) were females (Table 2). However, the presence of IgM without IgG antibodies was detected in 69 patients while the remaining 7 patients had both anti-DENV IgM and IgG. Among those with presumptive acute DENV infection, 29 (38.2%) had a confirmed acute DENV infection, all due to DENV-2. The occurrence of presumptive acute DENV infection was not associated with sex however, with respect to age, it was more likely to occur in children aged five years and above than in children under five years (OR 2.28, 95% CI: 1.35–3.86). This was also observed in children with confirmed acute DENV (OR 6.51, 95% CI: 1.78–23.87), Table 3. Although there were no significant differences in the occurrence of presumptive acute DENV infection between rainy and dry seasons, patients with confirmed acute DENV occurred more in dry season than in rainy season (OR 5.03, 95% CI: 1.68–15.01), Table 3.

Of patients with presumptive acute DENV infection, 30 (16.6%) had a duration of fever between 1–3 days whereas 43 (24.6) had 4–7 days. Patients with presumptive acute DENV presented with the following symptoms; lower respiratory tract symptoms 15 (19.7%), cough 14 (18.4%), vomiting 9 (11.8%), rashes 7 (9.2%), headache 7 (9.2%), abdominal pain 6 (7.9%), joint pain 3 (4.0%) and other symptoms 5 (6.6%), Table 2. The most reported symptoms other than fever in patients with confirmed acute DENV were vomiting 7 (24.1%), lower respiratory tract symptoms 5 (17.2%), headache 4 (13.8%), abdominal pain 4 (13.8%), cough each 4 (13.8%), rashes 3 (10.3%) and joint pain 2 (6.9%).

With regards to provisional diagnosis provided by the clinician, among those with presumptive acute DENV infection, 18 (23.7%) were malaria positive, 42 (55.3%) were considered as UTI cases, 16 (21.1%) pneumonia, 2 (2.6%) upper respiratory tract symptoms and 11 (14.5%) were diagnosed with other diseases (Table 2). A total of 10 patients were diagnosed with both malaria and UTI. On the other hand, the main diagnoses in patients with confirmed DENV were UTI 15 (51.7%), malaria 8 (27.6%), other diseases 5 (17.2%) and pneumonia 4 (13.8%). With respect to treatment given, 18 (23.7%) of those with presumptive acute DENV infection were prescribed with anti-malarial drugs and 68 (89.5%) were treated with antibacterials, 51 (67.1%) were prescribed with antipyretics while 11 (14.5%) were given other drugs (Table 2). It was noted that only 8 (10.5%) of patients with presumptive acute DENV were not prescribed with antibacterials. Additionally, most patients with confirmed acute dengue 24 (82.8%) were prescribed antibacterials while 8 (27.6%) were prescribed anti-malarial therapy, 22 (75.9%) antipyretics and 6 (20.7%) other drugs (Table 2).

Analysis of IgG antibodies indicated the presence of probable prior exposure to DENV in 24 (6.6%) of enrolled patients. Males were represented with a higher percentage than females (OR 0.38, 95% CI: 0.15–0.95). Similarly, the prevalence was higher in children aged above 5 (10.1%) than in children aged less than five years 8 (3.9%) i.e. OR 2.69, 95% CI: 1.10–6.56, Table 3. Most patients 18 (75.0%) had a history of fever within 4–7 days while 5 (20.8%) had fever for less than 3 days prior to recruitment.

### Chikungunya virus

It was observed that out of 364, 17 (4.7%) patients had anti-CHIKV IgM antibodies hence met the definition for presumptive acute CHIKV infection and of these, 8 (4.3%) were males and 9 (5.1%) were females. Among patients with presumptive acute CHIKV infection, 16 had IgM antibodies without IgG and 1 patient had both IgM and IgG antibodies. The presumptive acute CHIKV was found to be 5.4% (n = 11) in children under 5 years and 3.8% (n = 6) in children aged above 5 years as shown in Table 2. The duration of fever in patients with presumptive acute CHIKV was 1–3 days in 10 (58.8%) and 4–7 days in 7 (41.2%) of the patients. Our results show that occurrence of presumptive acute CHIKV was not related to patient's sex or age. Although the prevalence of presumptive acute CHIKV infection was 6 (3.3%) during rainy season and 11 (6.1%) in dry season, there were no significant differences in occurrence of presumptive acute CHIKV between the dry and rainy seasons (OR 1.85, 95%CI: 0.66–5.17), Table 3. The common reported symptoms among patients with presumptive acute CHIKV were cough, lower respiratory tract infections, rashes and abdominal pain (Table 2).

The provisional diagnoses of patients with presumptive acute CHIKV as provided by the clinician were as follows; malaria 2 (11.8%), UTI 10 (58.8%), pneumonia 7 (41.2%) and other diseases 1 (5.9%), Table 2. In addition, 2 patients were diagnosed with both malaria and UTI whereas 1 patient had malaria and other diseases. With regards to treatment given to patients, 15 (88.2%) of patients with presumptive acute CHIKV were treated with antibacterials while 2 (11.8%) were treated with anti-malarials. Furthermore, the present study has also revealed probable prior exposure to CHIKV infection in 5(1.4%) of all patients, of these 3(1.6%) were males and 2(1.1%) were females.

### Influenza virus, rotavirus and adeno virus

A total of 97 nasal swab specimens which were obtained from patients with nasal discharge were screened for influenza virus (Table 4). Out of 97 patients, 5 (5.2%) were positive for influenza virus, including two males and three females. Among those, the prevalence of influenza type B was 4 (4.1%) while influenza type A was 1(1%). In addition, 4 cases occurred in children above 5 years whereas 1 case was a child under the age of five years, as summarized in Table 5. Furthermore, 39 stool samples from diarrheic children were tested for rota-and adeno virus (Table 4). Our results show that among the 39 children, rotavirus was detected in 5 (12.8%) of patients while adenovirus was found in 2 (5.1%), Table 5.

**Table 4:** Demographic characteristic, enrollment temperature and duration of fever for patients tested for influenza, rota and adeno viruses.

Category	Subcategory	Influenza virus (N = 97)		Rota/Adeno virus (N = 39)	
		**n**	**%**	**n**	**%**
**Gender**	Male	43	44.3	21	53.8
	Female	54	55.7	18	46.2
**Age**	<5 years	73	75.3	27	69.2
	≥ 5 years	24	24.7	12	30.8
**Season**	Rainy	11	11.3	14	35.9
	Dry	86	88.7	25	64.1
**Temperature**	Mild	89	91.8	29	74.4
	Moderate	7	7.2	10	25.6
	High	1	1.0	0	0
**Duration of fever**	1–3 days	54	55.7	22	56.4
	4–7 days	42	43.3	17	43.6
	>7 days	1	1.0	0	0

**Table 5:** Proportions of febrile children with influenza, rota- and adeno-viruses.

	Influenza	Rotavirus	Adenovirus
**Category**	**n(%)**	**n(%)**	**n(%)**
Overall prevalence	5(5.2)	5(12.8)	2(5.1)
Male	2(4.7)	2(9.5)	1(4.8)
Female	3(5.6)	3(16.7)	1(5.6)
<5 years	1(1.4)	4(14.8)	1(3.7)
≥ 5 years	4(16.7)	1(8.3)	1(8.3)
Rainy	3(27.3)	0	0
Dry	2(2.3)	5(100)	2(8.0)

### Co-infections

Of 364 patients, 39 (10.7%) were found with multiple infections. The co-infection between malaria and presumptive acute CHIKV infection was 2 (0.6%). Thirty one (8.5%) of patients with presumptive acute DENV/confirmed acute DENV or probable prior DENV exposure infection were co-infected with malaria. In addition, 1 patient (0.3%) who was influenza-positive had a positive malaria blood slide. Co-infection between malaria and adenovirus infections was also detected in 1 patient (0.3%). Furthermore, 4 (1.0%) of the patients with presumptive acute CHIKV had a concomitant DENV infection.

## Discussion

We have demonstrated that DENV and CHIKV are among the viral etiologies of fevers in children in Kilosa district. To our knowledge, these diseases have neither been reported in this area nor have they been considered in the routine diagnosis of febrile patients in most health facilities in Tanzania. Our results have shown that many patients with malaria, presumptive acute CHIKV or DENV infections were clinically manifested by similar symptoms. Therefore, our findings point out the necessity of applying appropriate diagnostic tests in febrile patients.

In this study, we detected DENV, CHIKV, influenza virus, rota-and adeno virus in a considerable number of febrile patients. Our results showed that anti-CHIKV/DENV IgM antibodies were found in majority of patients within 7 days after the onset of fever hence suggesting possibility of acute infection (presumptive acute CHIKV/DENV infections). Normally, in acute CHIKV/DENV infections IgM antibodies appear first and can be detected during the first week of the disease particularly from day 5 while IgG antibodies appear shortly afterwards [38]–[40]. The detection of IgM antibodies in patients who had a history of fever for less that 5 days, possibly indicate a recent previous DENV exposure rather than acute infection. Since this study was cross-sectional, a cautious interpretation of this finding is necessary, particularly in the absence of comparison between acute and convalescent serum from the same patient.

In the present study we investigated IgG antibodies in order to make inference to previous exposure to DENV and CHIKV. Based on our data we have shown that some patients had previous exposure to DENV and CHIKV. This finding is important to understand the prevalence of these diseases in the study area. Previous studies have also indicated the occurrence of presumptive and prior exposure to CHIKV and DENV infections in mainland Tanzania and Zanzibar [6], [8], [41]. The prevalence reported here is almost similar with results obtained by Vairo et al (2014) for mainland Tanzania even though they reported a higher prevalence in Zanzibar [41]. Our results on previous exposure of some patients to CHIKV infection are however contrary to reports from previous studies conducted in northern regions of Tanzania and elsewhere [8], [42]. The discrepancy between the different studies may be attributed to geographical zones where the studies were done and target age groups.

The present findings revealed that both males and females were equally infected by DENV and CHIKV even though probable prior DENV exposure was observed more in males than in females. Other studies in Asia have reported dengue infections being higher in males than in females [43]–[45]. Furthermore, the comparison between age groups revealed that presumptive acute DENV infection and confirmed acute DENV was found more often in children aged five years and above than in children under five years. Similar findings were obtained from studies in Nicaragua, Central America where dengue infection was found predominantly in children 5–9 years old [46]. In this study, we observed that presumptive acute CHIKV/DENV infection did not vary with season while patients with confirmed acute DENV infection were more likely detected during the dry season. These findings suggest that the transmission of DENV was higher during the dry season, and agree with results from a previous study in northern Tanzania where more cases were more likely to occur during the dry season and another study in Indonesia which identified the occurrence of CHIKV all year round [8], [47]. Similarly, our findings concur with observations from previous studies in Vietnam and Thailand where high risk of dengue transmission was reported to occur during the dry season [48], [49]. However, other studies have demonstrated a high prevalence of CHIKV and DENV during a rainy season [50], [51]. The discrepancy between such findings and ours indicate the possibility of year round transmission of these diseases in our study area. A similar observation was also seen in malaria transmission pattern in the present study, in which the number of patients with malaria parasites was slightly higher during the dry season. Year round transmission pattern of vector-borne diseases may be due to presence of water bodies, such as rice paddies, irrigation canals, ponds and streams which favour breeding of mosquitoes during the dry season. Furthermore, only a few patients with presumptive acute dengue were detected during the dry season contrary to confirmed dengue. This finding implies that some patients with presumptive acute dengue could have had IgM antibodies from a previous recent dengue infection particularly those patients with fever of less than 4 days.

Our results revealed that most patients with presumptive acute CHIKV/DENV or confirmed acute dengue infection presented with similar symptoms as malaria. Similarly, no presenting symptoms distinguished patients with presumptive acute CHIKV infection from presumptive acute dengue or confirmed acute dengue infections. These findings are consistent with an earlier study conducted in northern Tanzania [8]. These results further confirm that that the absence of unique clinical manifestations among patients with these diseases poses a challenge in the diagnosis of such illnesses particularly in resource poor countries where diagnostic facilities are limited.

This study revealed the occurrence of influenza virus, rotavirus and adenovirus among the enrolled febrile children. Influenza virus was detected in 5.2% of patients slightly less compared to 8% obtained from National Sentinel Surveillance for influenza in Tanzania during 2008–2010 even though the prevalence of influenza type B in our study was high as compared to influenza type A [13]. Furthermore, we observed a low prevalence of rotavirus and adenovirus as compared to previous studies [7], [52]. It is possible that the inability to obtain fecal sample from other febrile children contributed to a low prevalence of rotavirus and adenovirus. However, these findings suggesting that these diseases should also be considered in the differential diagnosis of febrile patients.

The presence of multiple infections among febrile patients was evident in the present study. The findings revealed patients with malaria had a concomitant CHIKV, DENV, influenza or adenovirus infections. Concurrent DENV and CHIKV infections were also observed. Co-infections of malaria and dengue have also been reported from studies in Asia [53], [54]. A recent study in Tanzania reported co-infections with malaria in two thirds of the children which is higher than our findings [7]. With regards to co-infections between viral diseases, evidence from various studies in central Africa and Europe have demonstrated co-infections between CHIKV and DENV hence correlating with our findings [38], [50]. Co-infection is recognized as a major contributor to morbidity and mortality if left untreated [55]. Currently, mRDT is the only diagnostic test which is available in most health facilities in Tanzania [18]. These findings indicate that the diagnostic tools for discrimination of both malaria and other febrile diseases are crucial. Alternatively, patients with multiple infections will continue receiving incomplete therapy which will contribute to repeated visits to health facilities and possibly unnecessary deaths.

When comparing between the laboratory findings and the treatment prescribed by the clinician, we have shown that most patients with presumptive acute CHIKV/DENV or confirmed acute DENV infections were treated with antibacterials while only few patients were not treated with antibacterials. Currently, there is no specific medication for CHIKV or DENV infections, instead the use of antipyretics, pain relievers with fluid replacement therapy in case of dehydration is recommended [56], [57]. These findings confirm that shortage of diagnostic tests in developing countries is a major obstacle, which contributes to over-prescription of antimicrobials. If this challenge is not addressed, it will exacerbate the current problem of development of antibiotic resistance.

### Strengths and limitations

This study has reported viral etiologies of febrile illnesses in a district hospital which serves as a referral for primary health care facilities in the district and hence provides a good representation of the general population in the district. However, the specificity and sensitivity of the serological tests used in this study were not 100%. Therefore, cross-reactivity with other related viruses could occur. Furthermore, detection of IgM antibodies in acute serum without considering the convalescent serum could mislead the interpretation of results. For instance a negative IgM could not rule out a diagnosis of acute CHIKV or DENV infections since IgM may not be detected in samples collected in the very early stages of infection. In addition, IgM antibodies can persist in the body for about 2 months and thus a single rise of titer particularly in patients with history of fever for less than 5 days could indicate a recent past infection rather than an acute infection. Another limitation was our inability to perform PCR analysis for CHIKV due to lack of positive controls for the test.

## Conclusion

In this study, we intended to bring attention on the presence of viral etiologies of febrile illnesses in Kilosa district. Our results suggest that DENV, CHIKV, rota and adeno viruses should be considered in differential diagnosis of patients with fever. Furthermore, we found that these diseases were manifested with similar symptoms hence clinical diagnosis alone was not sufficient to discriminate them. The absence of diagnostic facilities was shown to be a major factor leading to improper management such that most patients were prescribed with unwarranted antibacterials. With a significant decline of malaria-related fevers in malaria endemic areas, these findings suggest that emphasis on non-malarial febrile illnesses is of paramount importance and this should be accompanied with the use diagnostic tools to enable proper diagnosis and management of febrile illnesses.

## Supporting Information

Table S1 **Sensitivity and specificity of the commercial laboratory test kits used to test DENV, CHIKV and Rota/Adeno viruses.**
(DOC)

Checklist S1 **STROBE checklist.**
(DOC)
