# Supplementary material for: Dengue and Chikungunya Fever among Viral Diseases in Outpatient Febrile Children in Kilosa District Hospital, Tanzania
Source: PLoS Negl Trop Dis. 2014 Nov 20;8(11):e3335. doi: 10.1371/journal.pntd.0003335 (PMC4239002; doi:10.1371/journal.pntd.0003335)
Supplement: Table S1 — Sensitivity and specificity of the commercial laboratory test kits used to test DENV, CHIKV and Rota/Adeno viruses. (DOC) [file pntd.0003335.s001.doc]

**Table S1.** Sensitivity and specificity of the commercial laboratory test kits used to test DENV, CHIKV and Rota/Adeno virus

| **Kit (Manufacturer)** | **Sensitivity** | **Specificity** | **Cross-reactivity** | **Source of information** |
| --- | --- | --- | --- | --- |
| **NovaLisa Dengue Virus IgM-ELISA.** (NovaTec Immundiagnostica GmbH, Germany) | 90% | 97.6% | There is a cross reactivity among flavivirus due to presence of common antigenic determinants. Bacterial contamination or freeze-thaw cycles of the specimen may affect the absorbance values | http://www.novatec-id.com/products/infectious-diseases/virology/dengue/ |
| **NovaLisa Dengue Virus IgG-ELISA.** (NovaTec Immundiagnostica GmbH, Germany) | >90% | 93% | There is a cross reactivity among flavivirus due to presence of common antigenic determinants. Bacterial contamination or freeze-thaw cycles of the specimen may affect the absorbance values | http://www.novatec-id.com/products/infectious-diseases/virology/dengue/ |
| **NovaLisa Chikungunya IgM μ-capture ELISA** (NovaTec Immundiagnostica GmbH, Germany) | >90% | >90% | No cross-reactivity was observed by using Rheumatoid factor-samples and samples containing antibodies against *Bordetella pertussis*, *Chlamydia trachomatis*, *Chlamydia pneumoniae*, Dengue Virus, TBE, *Helicobacter pylori*, HSV 2, Leishmania, Mycoplasma and Schistosoma. Cross reactivity with antibodies against *Borrelia*, CMV and Toxoplasma cannot be excluded | http://www.novatec-id.com/products/infectious-diseases/virology/chikungunya-new/ |
| **NovaLisa Chikungunya IgG capture ELISA** (NovaTec Immundiagnostica GmbH, Germany) | >90% | >90% | Samples with IgG antibodies against Dengue virus, Tick born encephalitis, CMV, EBV, and Helicobacter pylori showed no cross reactivity. Cross reactivity with antibodies against O’Nyong Nyong virus is not excluded | http://www.novatec-id.com/products/infectious-diseases/virology/chikungunya-new/ |
| RIDA® QUICK Rotavirus | 100% | 99% | A positive result does not rule out the presence of another infectious pathogen. A negative result does not necessarily rule out rotavirus or adenovirus infection | http://www.r-biopharm.com/wp-content/uploads/items/ridaquick-rotavirusadenovirus-combi-dipsticks-3855/N1002-RIDAQUICK-Rota-Adeno-Box-10-08-10_GB.pdf |
| RIDA® QUICK Adenovirus | 90% | 100% | A positive result does not rule out the presence of another infectious pathogen. A negative result does not necessarily rule out rotavirus or adenovirus infection | http://www.r-biopharm.com/wp-content/uploads/items/ridaquick-rotavirusadenovirus-combi-dipsticks-3855/N1002-RIDAQUICK-Rota-Adeno-Box-10-08-10_GB.pdf |
